# Filamentous bacteriophages: impact on prokaryotic virulence, eukaryotic immunity, and implications for phage therapy

**DOI:** 10.3389/fimmu.2026.1904873

**Published:** 2026-09-23

**Authors:** Ambarish C. Varadan, Juris A. Grasis

**Affiliations:** 1 Department of Microbiology and Immunology, Emory University, Atlanta, GA, United States; 2 Department of Molecular and Cell Biology, University of California, Merced, Merced, CA, United States; 3 Quantitative and Systems Biology Graduate Group, University of California, Merced, Merced, CA, United States; 4 Health Sciences Research Institute, University of California, Merced, Merced, CA, United States

**Keywords:** adaptive immunity, bacteriophage, bacteriophage–host interactions, filamentous, immunogenicity, immunomodulation, innate immunity, *Inoviridae*

## Abstract

Filamentous bacteriophages, of the family *Inoviridae*, are characterized by their reproduction and secretion by their bacterial hosts without killing them. These viruses have evolved mutualistic relationships with their bacterial hosts by impacting bacterial phenotypes in a variety of ways, including biofilm formation, horizontal gene transfer, colonization, and motility. Previous studies have reported that filamentous bacteriophages contain virulence factors that promote the pathogenesis of bacterial infections in animal models and are associated with exacerbating infections in humans. Additionally, these phages have been experimentally used as cloning vectors for DNA sequencing, phage display, and nanotechnological applications. Finally, viromic studies have revealed a substantial presence of filamentous bacteriophages in the human gut. However, their interactions with mammalian cells have not been extensively explored. Here, we review the immunogenicity of filamentous bacteriophages in eukaryotic cells. First, we address how filamentous bacteriophages impact the fitness of their prokaryotic hosts. Then, we review their immunogenicity by focusing on the direct interactions between filamentous bacteriophages and eukaryotic cells that may indicate how these viruses influence various functions. Finally, we provide some thoughts on their impact for use in therapeutics. Through their ubiquitous interactions with prokaryotic and eukaryotic cells, there is still much to be learned about how filamentous bacteriophages affect the biology, pathogenesis, and physiology of the human gut. Unraveling these interactions will provide further evidence as to the importance of the virome to human health.

## Introduction

Bacteriophages colonize virtually all niches of the human body, including skin (1), oral cavity (2), lungs (3), urinary tract (4), and are most abundant in the gastrointestinal tract (5). Within the gut, bacteriophages comprise a major component of the virome, which also includes eukaryotic viruses, viruses that infect other microorganisms such as fungi and archaea, and plant viruses that are primarily derived from diet and environmental exposure (6). The human intestinal virome is established at birth and is initially dominated by bacteriophages, whereas eukaryotic viruses gradually emerge after birth, as measured in meconium samples (7, 8). Although research on the microbiome has largely focused on its bacterial constituents, increasing evidence indicates that the virome also plays an important role in human health and disease. Alterations in virome composition have been reported in many pathologies, including colorectal cancer (9, 10), inflammatory bowel disease (11–14), obesity (15), type I diabetes (16), and nonalcoholic fatty liver disease (17), to name just a few. Although it remains unclear whether these alterations are one of the causes or a result of the abovementioned multifactorial pathologies, studies have shown that the enteric virome contributes to inflammation (18–20) and is a key player in influencing long-term health outcomes through shaping enteric microbial communities (21), modulating immune responses (19), and regulating metabolism (22).

Despite their abundance, the direct interactions between bacteriophages and their human hosts remain relatively understudied. Although bacteriophages cannot directly infect human cells, they can influence host physiology indirectly through their effects on bacterial communities and directly through interactions with mammalian cells. Bacteriophage-mediated lytic activity clears pathogenic bacteria at sites of infection (23) and selects for bacterial colonizers at mucosal sites (24). However, some bacteriophages encode toxins that are released during bacterial lysis, leading to severe disease states (25). Most bacteriophages in the human gut are nonenveloped DNA viruses, either belonging to the class Caudoviricetes with double-stranded DNA (dsDNA) genomes or to the class Microviricetes with single-stranded DNA (ssDNA) genomes (26).

Recent characterization of filamentous bacteriophages from human commensal bacteria has expanded the known diversity of the intestinal virome (27). Filamentous bacteriophages are a subgroup of *Inoviridae*, a family of nonenveloped ssDNA bacteriophages. They infect both Gram-positive and Gram-negative bacterial species, and even some species of archaea (28). These bacteriophages exhibit a lifestyle distinct from virulent and temperate phages. Virulent bacteriophages replicate through a lytic lifecycle that culminates in host bacterial cell lysis, whereas temperate bacteriophages follow a lysogenic lifecycle in which they can either integrate into the host bacterial chromosome as a prophage or exist as an episome. Upon exposure to specific inducers, these temperate phages are activated, leading to the lytic cycle and subsequent lysis of the host bacteria (29).

In contrast, filamentous bacteriophages establish chronic lifecycles wherein progeny virions are continuously extruded through the bacterial cell envelope without lysing their host (30). Some filamentous bacteriophages, including the Ff phages M13, Fd, and f1, persist as episomes, while others, such as Pf4, MDAϕ, and CTXϕ, integrate into the bacterial chromosome as temperate-like bacteriophages, however, they do not lyse their hosts. Like other temperate phages, chromosomally integrated filamentous bacteriophages can confer advantageous traits to their bacterial host that influence colonization (31), virulence (32), and nutrient acquisition (33). Due to their impact on bacterial phenotypes, some filamentous bacteriophages have been considered as virulence factors that promote the pathogenesis of bacterial infections and are associated with adverse health outcomes in humans (34, 35).

This minireview focuses on the interactions of filamentous bacteriophages with both bacterial and mammalian cells. We first discuss their effects on bacterial fitness and virulence, followed by an overview of emerging evidence describing their direct interactions with mammalian cells and the potential consequences for host health and physiology. Because much of the current understanding of filamentous phage biology derives from studies of the Ff phages of *Escherichia coli* and the Pf phages of *Pseudomonas aeruginosa*, these systems are the primary focus of this review. The evidence provided by these studies sheds light on the complex and dynamic interactions between filamentous bacteriophages and mammalian cells and encourages exploration of more filamentous bacteriophages at these sites of interaction.

## Effects of filamentous phages on bacterial cells

### Impact of episomal filamentous bacteriophage on bacterial fitness

Filamentous bacteriophage infection is highly productive, with Ff phages generating approximately 1,000 virions per infected cell per generation. This imposes an immense metabolic burden on the host, impairing host protein synthesis and RNA expression (36) and inducing envelope stress (37). Disruption of phage secretion through inactivation of proteins required for assembly complex formation or initiation of virion assembly results in host-cell death due to the accumulation of cristae-like invaginations within the inner membrane (38). Interestingly, mutation of the assembly protein pI does not produce the same lethal phenotype, suggesting that bacterial viability depends on maintaining a balance between phage synthesis and virion assembly (36).

Infection of *Escherichia coli* by bacteriophage M13 has been shown to damage lipopolysaccharide (LPS) and promote its release from the bacterial outer membrane (37). Combined with the energetic demands associated with phage protein production and secretion, these effects contribute to reduced bacterial growth. Although infected bacteria remain viable and exhibit no significant changes in cellular dimensions, they display altered colony morphology characterized by smaller, more transparent colonies (39). Beyond the expected upregulation of phage genes, host transcriptional profiles are rapidly altered following infection (40). One of the host genes that is greatly upregulated in response to Ff infection is the phage shock protein (psp), pspA (41). Phage shock proteins are a type of extracytoplasmic stress response (ESR) that help to maintain the integrity of the bacterial cell envelope under stressful conditions (42). Ff phage infection induces pspA expression due to the phage protein secretin pIV localizing within the *E. coli* envelope (43). These phage secretins have a unique ability to insert into either the outer or inner membrane of Gram-negative bacteria (44). PspA maintains the proton motive force (PMF) by preventing proton (H^+^) leakage across the damaged cytoplasmic membrane (45).

Homologs of the psp system are present in both Gram-positive and Gram-negative bacteria. In addition to playing crucial roles in the virulence of intracellular pathogens *Salmonella typhimurium* (46) and *Shigella flexneri* (47), the psp response is important for biofilm formation in *E. coli* strain K-12 (48) and has also been linked to the formation of *E. coli* persisters, which are multidrug-resistant phenotypic variants linked to chronic infections (49). Biofilms are highly ordered structures characterized by the production of an extracellular polymer biofilm matrix (50). Notably, Secor et al. (32) reported that bacteriophage Fd had biofilm-building capability as it formed highly organized structures when mixed with Hyaluronan (HA), a glycosaminoglycan present in the airways of patients with Cystic Fibrosis (CF). These findings suggest that filamentous phages may contribute directly to biofilm architecture in addition to influencing bacterial stress responses. Future studies examining the relationship between Ff phage infection, Psp signaling, biofilm development, and antibiotic tolerance may provide important insights into the contribution of filamentous phages to *E. coli* persistence and pathogenesis.

### Impact of filamentous bacteriophage on bacterial fitness

Filamentous bacteriophages integrate into the chromosomes of their bacterial hosts and function as mobile genetic elements that facilitate the acquisition of new traits (51). In the context of human disease, these traits include biofilm formation (32), evasion of host immune responses (35), coding for resistance to antibiotics (32), and acquisition and utilization of certain nutrients (33). One of the best characterized examples is CTXϕ, a temperate-like filamentous bacteriophage of *Vibrio cholerae*. CTXϕ carries the *ctxAB* genes, which encode the A and B subunits of cholera toxin and can transduce *V. cholerae* strains from non-toxigenic to toxigenic (52). The disease is caused by the secretion of cholera toxin, which provides an advantage to the bacterial host as it promotes diarrhea in humans, which results in the dissemination of the bacterial pathogen (31). Another well-studied temperate-like filamentous phage is MDAϕ of *Neisseria meningitidis*, which enhances bacterial colonization of nasopharyngeal FaDu cells (30). Bille et al. reported that bacteria in the upper layer of the biomass produced large quantities of MDAϕ particles that remained attached to the bacterial cell wall, leading to the formation of bundles that stabilized bacteria-bacteria interactions. This could, in turn, increase the frequency of bacterial dissemination in the bloodstream (31). Entry of MDAϕ into bacterial cells requires Type IV pili, structures that are also essential for blood vessel colonization during meningococcal pathogenesis (53). More recently, Mouville et al. reported that MDAϕ preferentially binds positively charged Type IV pilin expressed by hyper adhesive bacterial variants, thereby promoting their selection and increased colonization efficiency (54).

The contribution of filamentous phages to bacterial fitness is particularly evident in *Pseudomonas aeruginosa*, which harbors a group of related filamentous phages called the “Pf1-like” phages: Pf1, Pf4, Pf5, Pf6, and Pf7. The pathogenicity of *P. aeruginosa* is closely linked to its ability to form biofilms, and multiple studies have reported increased expression of Pf phage genes during biofilm growth (55, 56). Furthermore, biofilms can produce exceptionally high concentrations of Pf4, reaching up to 10^11^ PFU/mL (57). Pf4 contributes to biofilm formation by inducing cell death and increasing the frequency of small colony variants (SCVs), which have been detected in patients with cystic fibrosis and correlate with poor pulmonary function and increased antibiotic resistance (58). Secor et al. reported that Pf4 assembled the *P. aeruginosa* biofilm matrix into highly ordered liquid crystalline structures that enhanced biofilm adhesion and increased tolerance to desiccation and antibiotics (32). These findings highlight the capacity of filamentous phages to influence not only bacterial gene content but also the physical properties of biofilms that contribute to chronic infection.

Beyond biofilm formation, Pf phages are implicated in the progression of chronic wound infections. *P. aeruginosa* is frequently isolated from chronic wounds (59, 60) and is associated with delayed wound healing, especially in chronic skin injuries (61, 62). Sweere et al. reported that wounds colonized with Pf-positive strains of *P. aeruginosa* were significantly slower to heal than those colonized with Pf-negative strains (35). Furthermore, Pf reduced the number of bacterial inoculum required to establish infection and increased *P. aeruginosa*-induced morbidity and mortality in mice. Re-epithelialization, a critical step in wound repair, depends on keratinocyte migration and proliferation (63). Pf suppressed the expression of chemokines CXCL1, CCL7, and CCL22 in keratinocytes, impairing keratinocyte migration and delaying re-epithelialization (64).

Collectively, these studies demonstrate that filamentous bacteriophages can profoundly influence bacterial fitness and pathogenicity by promoting toxin production, enhancing colonization, stabilizing biofilms, and facilitating chronic infection. In the following section, we examine the direct interactions between filamentous bacteriophages and mammalian cells and discuss the mechanisms through which these viruses modulate host immune responses.

## Effects of filamentous bacteriophages on eukaryotic immunity

Although bacteriophages do not infect eukaryotic cells due to a lack of phage-specific receptors on the cell surface (65), they are a component of the resident microbiota and colonize virtually all niches of eukaryotic organisms (1–5). Furthermore, due to phage ubiquity in a variety of environments (66–69), eukaryotic organisms are constantly exposed to bacteriophages. Thus, eukaryotic organisms, including humans, may be considered long-term hosts to diverse communities of phages that colonize the luminal and mucosal sites (70–72). Therefore, there is increasing interest in the direct impact of bacteriophages on human cells and the cellular response to these direct phage interactions. This has been driven, in part, by efforts to expand our understanding of endogenous bacteriophages residing on and within the human body (73), but also to develop phage therapies to target antibiotic-resistant bacterial infections (74) and tumors for cancer immunotherapy (75, 76). In this section, we examine the immunogenicity of filamentous bacteriophages by focusing on their direct interactions with mammalian cells. As bacteriophages are most abundant in the gastrointestinal tract, we will first discuss what is known about the interactions between filamentous bacteriophages and intestinal epithelial cells. Next, we address some of the mechanisms by which innate immune cells recognize and respond to filamentous bacteriophages. We will conclude by reviewing adaptive immune responses to filamentous bacteriophages.

### Interaction between filamentous bacteriophages and intestinal epithelial cells

Although the GI tract represents the largest reservoir of bacteriophages in the human body (5, 77), very few studies have examined whether bacteriophages can directly stimulate intestinal epithelial cells (**Figure 1**). We previously reported that M13 induced proinflammatory IL-8 in intestinal epithelial HT-29 cells at both transcript and protein levels (**Figure 1A**) (78). This response was dose-dependent and greater than those induced by Fd (**Figure 1B**) or by lytic bacteriophages T4 and ϕX174. As IL-8 is a potent neutrophil chemoattractant produced by intestinal epithelial cells (79), these findings suggest that filamentous bacteriophages may contribute directly to mucosal immune responses. Elevated IL-8 expression has been reported in the inflamed mucosa of patients with ulcerative colitis (80–82), highlighting the potential relevance of bacteriophage-induced epithelial signaling in intestinal inflammation.

**Figure 1:**
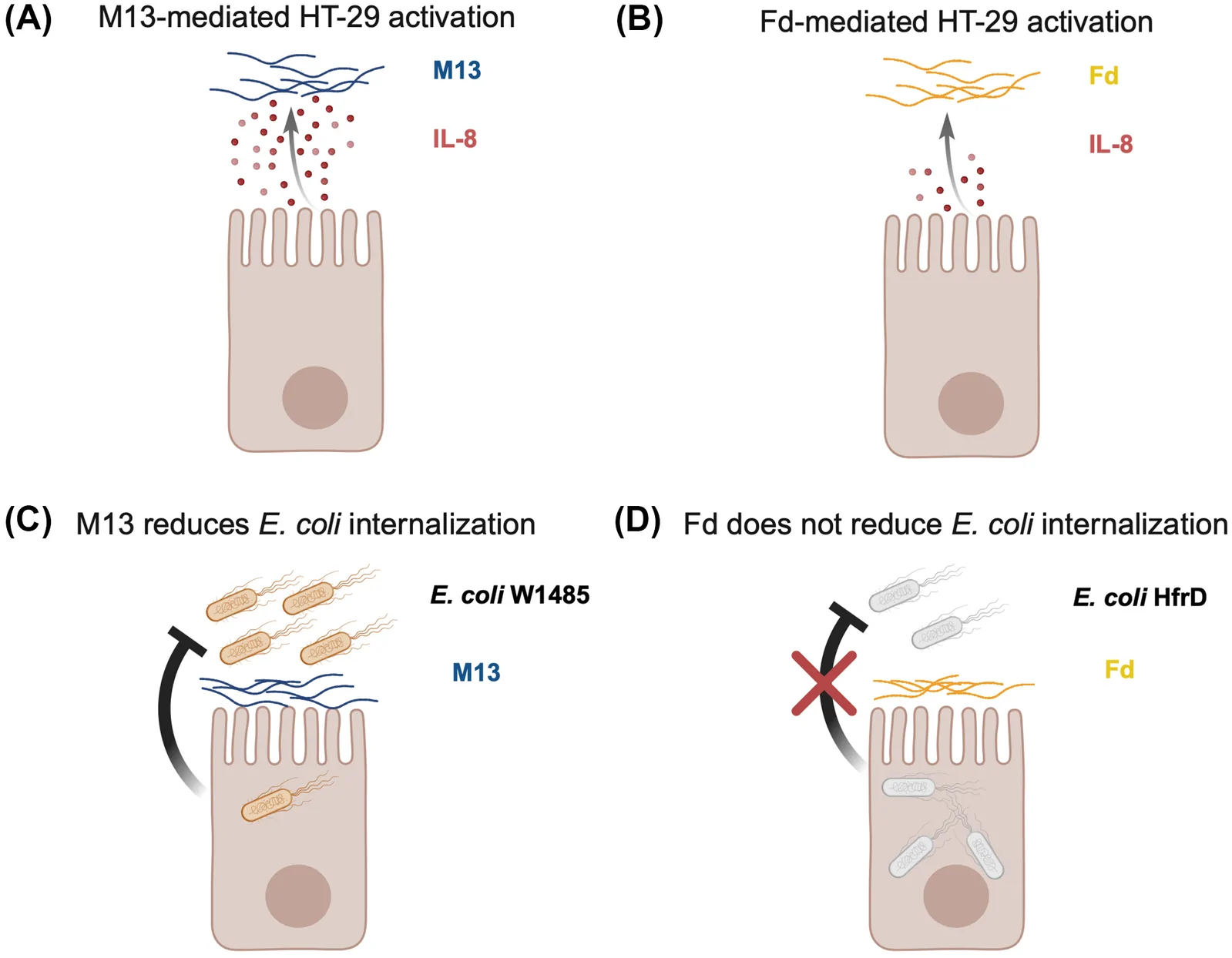
Direct and tripartite interactions between filamentous bacteriophages M13 and Fd, their host bacteria, and intestinal cells. Models depicting direct activation of HT-29 cells in response to M13 **(A)** and Fd **(B)**. M13 induced greater IL-8 secretion and expression than Fd, demonstrating that closely related bacteriophages can elicit differential responses in the same cell type. Models depicting tripartite interactions between M13 **(C)** and Fd **(D)** filamentous bacteriophages, their host bacteria, and intestinal epithelial cells. **(C)** M13 impacts the internalization of *E. coli* W1485 (host of M13) by HT-29 cells. **(D)** Fd fails to impact internalization of *E. coli* HfrD (host of Fd) by HT-29 cells. Pre-stimulating HT-29 cells with M13 prior to infection with *E. coli* W1485 resulted in internalization of fewer bacterial cells (78). In contrast, pre-stimulating HT-29 cells with Fd before infection with *E. coli* strain HfrD did not reduce bacterial internalization, again demonstrating differential immunogenicity in response to related bacteriophages.

Based on a previous study by Barr et al. that demonstrated that the adherence of lytic bacteriophage T4 to the mucus layer of tissue-culture cells reduced bacterial colonization and infection (24), we wanted to evaluate whether filamentous bacteriophages M13 and Fd would impact bacterial internalization by HT-29 cells. We observed that co-stimulating HT-29 cells with M13 and *E. coli* W1485 (host of M13) significantly reduced the number of bacteria internalized by the intestinal epithelial cells (**Figure 1C**). Furthermore, pre-stimulating HT-29 cells with M13 prior to infection with *E. coli* W1485 also led to the internalization of fewer bacteria (78). In contrast, pre-stimulating HT-29 cells with Fd prior to infection with *E. coli* strain HfrD (host of Fd) did not result in decreased bacterial internalization (**Figure 1D**). As both W1485 and HfrD are commensal strains of *E. coli*, it could not be concluded that M13 protects HT-29 cells against bacterial invasion. To make these conclusions, internalization studies should be conducted with enteric bacterial pathogens to evaluate the protective potential of M13. If M13 can indeed protect HT-29 cells against pathogenic bacterial invasion, the mechanism(s) underlying M13-mediated reduction of bacterial internalization can then be determined.

Sweere et al. (35) reported that monoclonal antibodies generated against the Pf4 major capsid protein CoaB reduced the incidence of *P. aeruginosa* wound infections as well as promoted the phagocytic engulfment of PAO1 (a strain of *P. aeruginosa* carrying Pf4). Therefore, antibody-mediated recognition of Pf4 promotes *P. aeruginosa* phagocytosis. Given that the major capsid protein pVIII is the most abundant protein on the surface of M13, it would be interesting to determine whether targeting the major capsid protein of M13 would affect bacterial internalization by HT-29 cells. Our results suggest that M13 and Fd may employ potentially distinct interactions with colonic epithelial cells. The reason(s) behind this differential interaction may be related to differences in their respective capsid protein structure or amino acid composition. Although M13 and Fd are structurally very similar (30), solid-state NMR spectroscopy has revealed that the major capsid protein pVIII in M13 and Fd differs by a single amino acid; an uncharged Asparagine at position 12 in M13 corresponds to negatively charged Aspartate in Fd (83). The loss of a single negative charge in M13 can cause the Lysine residue in position 8 (Lys8) to adopt a different conformation and form salt bridges with Aspartic acid residues in positions 4 and 5 (Asp4 and Asp5), thereby increasing its rigidity compared to that of Fd. As this motif is repeated more than a thousand times throughout the major capsid protein of the bacteriophage, altering electrostatic and hydrogen-bonding interactions between residues might very likely play a significant role in the differential immunogenicity to these bacteriophages.

We also reported that M13 induced antiviral Type I and Type III IFN expression in HT-29 epithelial cells, supporting previous studies demonstrating that filamentous bacteriophages can trigger Type I IFN production in phagocytes (35). Single-cell RNA sequencing revealed that Pf4 upregulated several antiviral response genes in human basal epithelial cells (BCs), including IRF7 (84), a key transcription factor downstream of TLR3/TRIF signaling primarily induced by Type I IFNs and viral sensing (85). Type I IFN signaling elicits antiviral responses against viral pathogens through the induction of interferon-stimulated genes (ISGs) (86, 87), which target multiple aspects of the viral infectious cycle such as viral attachment (87) and entry (88), viral gene expression (89) and protein translation (90), to name just a few. Although filamentous bacteriophages have been reported to induce Type I IFN responses (78, 84), the impact of these antiviral responses on bacteriophages has not been studied and could be a key component of immunological tolerance (91), which will be covered in a later section. In addition to inducing antiviral immunity, Type I IFN signaling also plays a role in antibacterial immunity (92, 93), and M13-mediated Type I IFN induction may contribute to the observed reduction in bacterial internalization by HT-29 cells. Comprehensive transcriptomic analyses of bacteriophage-stimulated intestinal epithelial cells will be valuable for defining both the signaling pathways activated by filamentous phages and the mechanisms through which epithelial cells detect them.

Bacterial pathogens have evolved multiple strategies to colonize and invade intestinal epithelial cells (IECs) (94) and assessing internalization alone does not provide a comprehensive picture, as it does not account for bacterial colonization on the epithelial surface. Given the number and diversity of bacteriophages in the human gut (95), further studies are needed to explore the interactions between bacteriophages and the epithelium. In a recent study using an *in vitro* Caco-2/TC7 intestinal epithelial cell model, Douadi et al. reported that M13 exhibited higher cellular association compared to lytic bacteriophages T4 and ΦX174 (96), suggesting that interaction with cellular moieties may differ between different bacteriophages. They also showed that the cellular-associated M13 particles were localized with actin of the microvilli on the apical surface. The mechanism(s) underlying this localization of M13 are unknown, but one possibility could be a specific interaction between bacteriophage capsid proteins and actin leading to the enrichment of M13 particles at the microvilli. M13 has previously been shown to increase gene expression of integrins when interacting with human prostate cancer cells (97). Protein-protein interactions between M13 and actin could indicate an active process leading to bacteriophage localization at the apical surface of intestinal epithelial cells.

Alternatively, filamentous phages may exert direct intracellular effects. In the above-mentioned study, Douadi et al. also observed internalization of M13 by intestinal epithelial cells. M13 has been reported to enter epithelial cells through clathrin-mediated endocytosis and macropinocytosis (98). Given that both filamentous bacteriophages (35, 96, 98) and commensal bacteria are internalized by human cells (99), it is also likely that internalized bacteriophages could regulate bacterial populations intracellularly (99) through mechanisms that operate independently of a mucus barrier.

Collectively, these studies demonstrate that filamentous bacteriophages are not passive components of the intestinal virome. Rather, they can directly stimulate proinflammatory and antiviral responses in intestinal epithelial cells and alter host–bacterial interactions. These findings support the emerging view that filamentous bacteriophages represent active participants in host–microbe communication within the gastrointestinal tract.

### Innate immune sensing of filamentous bacteriophages

Underneath the intestinal epithelium lies the lamina propria, where sentinel immune cells reside to sample the gut environment. Among these, macrophages and dendritic cells (DCs) play crucial roles in mediating innate immune responses in the mucosa (100, 101). Douadi et al. observed that bacteriophages M13, T4, and ΦX174 translocated across intestinal epithelial cells in both *in vitro* and *in vivo* models (96), raising the possibility that bacteriophages residing within the human gut directly interact with underlying innate immune cells. Consistent with this idea, studies have shown that bacteriophages can directly induce both pro-inflammatory and anti-inflammatory responses (102) in human peripheral blood mononuclear cells (PBMCs), with the nature of these responses depending on both the phage and cell in question. TNF is critical to phagocytosis and bacterial clearance (103). Sweere et al. reported that Pf4 decreased TNF production in murine bone marrow-derived dendritic cells (BMDCs), macrophages, and human U937 and primary macrophages, which resulted in reduced clearance of *P. aeruginosa* by these cells (35). In contrast, Fd did not reduce TNF production or impair bacterial phagocytosis in murine BMDCs, suggesting that filamentous bacteriophages can elicit markedly different immune responses in the same cell type. Although the basis for these differences remains unclear, variation in capsid structure or virion composition may contribute to their differential immunogenicity.

In addition to evaluating cytokine responses in immune cells, the ability of filamentous bacteriophages to recruit immune cells has also been studied. Due to its role in promoting bacterial infection, Sweere et al. investigated whether Pf4 could also inhibit leukocyte recruitment, thereby increasing bacterial pathogenesis. However, they observed that the presence of Pf in wound inoculations did not significantly impact leukocyte recruitment to sites of infection (35). In a later study by Bach et al, they reported that Pf directly inhibited chemokine CXCL1 expression during inflammatory stress to limit HaCaT keratocyte cell migration and delayed wound healing (64). Furthermore, Eriksson et al. observed that neutrophils stimulated with media from M13-stimulated Tumor-associated macrophages (TAMs) did not exhibit increased migration (104). These studies serve to highlight the diversity of immune responses elicited by filamentous bacteriophages in different cell types.

Mechanisms underlying innate immune cell recognition of filamentous bacteriophages have been explored. Pf4-mediated suppression of TNF and phagocytic capacity was abolished in phagocytes from TRIF^−/−^ mice but remained intact in MyD88^−/−^ cells, indicating that this response depended on TRIF-mediated signaling pathways (35). Furthermore, Pf4-induced TNF suppression was associated with TLR3-TRIF-mediated Type I IFN production in murine BMDCs, resulting in impaired bacterial clearance. These findings demonstrate that filamentous bacteriophages can induce antiviral signaling programs that paradoxically compromise antibacterial immunity and enhance pathogen persistence.

In the same study, Sweere et al. observed that Pf4 was endocytosed by multiple PBMC populations, including monocytes, DCs, and B cells (35). Additional evidence for innate immune recognition of bacteriophages was reported by Gogokhia et al., who reported that pre-treatment of specific pathogen-free mice with a bacteriophage cocktail enhanced IFNγ production by CD4+ T cells following stimulation with BMDCs (105). Interestingly, this response was maintained in TLR3^−/−^ DCs but was abolished in TLR9^−/−^ DCs, suggesting that TLR9-dependent recognition contributes to bacteriophage-induced activation of adaptive immune responses (105).

Finally, filamentous bacteriophages can also impact immune cell polarization. *P. aeruginosa* PAO1 superinfected with Pf4 caused increased expression of genes associated with the anti-inflammatory M2 macrophage phenotype in murine bone marrow-derived macrophages (BMDMs), including IL-10 and Arg1 (106).

Collectively, these studies demonstrate that filamentous bacteriophages can directly activate immune cells through regulating cytokine production, altering phagocytic activity and polarization, and shaping downstream signaling pathways.

### Adaptive immune responses to filamentous bacteriophages

Much of our knowledge of adaptive immune responses to bacteriophages has come from phage therapy studies in humans and mice. Bacteriophages have been administered via various routes (107), including oral (108, 109), intravenous (110), nasal (111), and topical (112). The route of administration can be impacted by a variety of factors such as phage size, phage morphology, cell types present at the site of administration, and primary mode of infection (107, 113, 114). Hence, these factors need to be considered when designing experiments evaluating the efficacy of phage therapy. Intravenous administration enables systemic delivery of bacteriophages and achieves higher bioavailability compared to oral, nasal, or topical routes (107). However, this method of delivery also leads to rapid clearance of bacteriophages from blood (110, 115). Bruttin et al. reported that oral administration of bacteriophage T4 did not significantly increase T4-specific antibodies in patient serum (108). This could have been potentially due to the very low doses of bacteriophages administered due to safety concerns or the lack of an adjuvant. In contrast, Majewska et al. reported that long-term oral administration of T4 in mice elicited humoral responses, with IgG detected in the blood on day 36, and IgA detected in feces on day 79 (109). The magnitude and timing of this response were dependent on the phage dose and duration of exposure.

Gogokhia et al. reported that the administration of a bacteriophage cocktail to specific pathogen-free (SPF) mice led to increased CD4+ and CD8+ T cells within mesenteric lymph nodes (mLNs). Furthermore, they showed that although bacteriophages did not directly cause increased IFNγ production in CD4+ T cells, DCs that were pre-incubated with bacteriophages increased the production of IFNγ in CD4+ T cells (105). These findings suggest that bacteriophage-mediated CD4+ T cell activation occurs indirectly through antigen-presenting cells rather than through direct stimulation of lymphocytes. While these studies primarily used lytic bacteriophages, the abundance of filamentous bacteriophages in the human gut (27), coupled with their transcytosis across the epithelial barrier (96), would seem to suggest that they could also potentially induce humoral responses. Further evidence to support this idea comes from Gaubin et al., who reported that Fd was taken up by human B cells into endosomal compartments prior to being targeted to MHC I and II processing pathways (116). They also showed that CD8+ CTLs primed with Fd displaying an exogenous peptide exhibited cytotoxic activity and lysed Epstein-Barr virus-infected B cells. Although *in vitro* priming with wild-type Fd did not elicit cytotoxic responses, this demonstrates that filamentous bacteriophage-derived antigens can be presented to T cells by APCs. Sweere et al. reported that BMDCs pre-treated with monoclonal antibodies against the major capsid protein (CoaB) of Pf4 were able to effectively phagocytose *P. aeruginosa* (45). In the same study, they immunized mice with a peptide version of this epitope and were able to successfully generate humoral immunity against Pf phage. Vaccinating mice with monoclonal antibodies targeting CoaB reduced the incidence of *P. aeruginosa* wound infections, suggesting that humoral immunity against Pf is protective against *P. aeruginosa* pathogenesis (45).

### Immunological tolerance to bacteriophages

While some studies have shown that specific bacteriophages elicit stimulatory (78, 84) or immunosuppressive (35, 64) responses, others have reported that bacteriophages do not induce stimulatory responses in mammalian cells (117–119). Given the diversity of bacteriophages in mammalian systems (95, 120), in the context of immunogenicity, the implications from these studies are: (1) the impact of bacteriophages on mammalian cells is both phage- and cell-specific and (2) the mammalian system reacts to specific bacteriophages while simultaneously tolerating most of them (70). Immunological tolerance and clearance are key issues to consider when evaluating specific bacteriophages for therapeutic use. Douadi et al. observed that bacteriophages T4, M13, and ΦX174 translocated across intestinal epithelial and endothelial cells without inducing pro-inflammatory responses, cytotoxicity, or disrupting barrier integrity. While we showed that M13 induced pro-inflammatory responses in intestinal epithelial HT-29 cells (78), these differences may be due to the different cell lines that were used, as HT-29 and Caco-2/TC7 cells exhibit distinct epithelial phenotypes (96). Furthermore, they observed that M13 was endocytosed by Human Umbilical Vein Endothelial Cells (HUVECs) and evaded lysosomal degradation even after 24 hours post-incubation (96). Previous studies have also reported prolonged accumulation of bacteriophages within human cells (114, 121, 122). Lehti reported that approximately 30% of bacteriophage PK1A2 particles that were internalized by human neuroblastoma SK-N-SH cells remained infectious 24 hours after being internalized (122). Bichet et al. reported that lung epithelial A549 cells, HUVECs, and human foreskin fibroblast BJ cells, all internalized bacteriophage T4, and the majority of internalized phage particles remained active and stably persisted within the cells for up to 18 hours (114). These results underscore the importance of two key aspects of bacteriophage immunogenicity: tolerance and clearance. The efficacy of phage therapy depends, in part, on the ability of the host to maintain immunological tolerance to therapeutic bacteriophages while also being able to mount a robust immune response to the bacterial infection. It is also dependent upon the precise delivery of therapeutic bacteriophages to the site of infection to kill bacterial pathogens. However, the uptake and subsequent clearance of bacteriophages by multiple cell types (96, 114, 121, 122) can impede the success of phage therapy. In the next section, we discuss the implications of filamentous bacteriophage immunogenicity on therapeutic applications of bacteriophages.

Thus far, we have discussed several studies that have explored whether and how bacteriophages can induce or suppress immune responses in mammalian cells. In the following section, we discuss the impact of these immune responses on bacteriophages and their implications for phage therapy.

## Implications for therapeutics

Phage therapy is the administration of virulent phages directly to a patient to lyse the bacterial pathogen causing a clinically problematic infection (74). Although filamentous bacteriophages have not yet been used in phage therapy, as they do not lyse their bacterial hosts, their immunogenicity has important implications for phage therapy. Collectively, studies have shown that bacteriophage immunogenicity is both phage- and cell-type dependent. This variability underscores the critical need to evaluate the many aspects of phage-mediated immunogenicity, such as uptake (35, 96, 98, 114), entry (96, 98), kinetics and dose-dependency of activation (35, 78), suppression (35), recruitment of immune cells (35, 64), and induction of humoral immune responses (35, 105). Delineating this multitude of processes would provide a holistic picture of how human cells respond to bacteriophages, as well as help to understand how to effectively utilize bacteriophages to harness the power of the immune system. This would, in turn, be informative when designing phage-based strategies to combat multidrug-resistant (MDR) pathogens. As an example, Roach et al. reported that *P. aeruginosa* lung-infected WT mice depleted of their neutrophils were completely unresponsive to inhaled phage PAK_P1 treatment and succumbed to *P. aeruginosa* infection. Successful clearance of the pathogen required synergy between murine neutrophils and the administered phages (23). In addition, they showed that phage therapy was effective in mice lacking innate and adaptive lymphoid cells but ineffective in mice lacking innate immune activation, demonstrating the specificity of synergy to neutrophils. Furthermore, the administered phages were immunologically well tolerated and not cleared by host immune responses (23). This group coined the term “immunophage synergy” to describe how the host immune response can complement phage therapy in successfully eradicating infection, particularly phage-resistant bacterial strains. However, anti-phage immunity can also impede the efficacy of phage therapy. Berkson et al. reported that antibodies generated in response to a phage cocktail strongly neutralized the bactericidal activity of the myophages (Φ19 and Φ53) used in the phage cocktail. They also observed that anti-phage neutralizing antibodies accelerated bacteriophage clearance from murine tissues and reduced the efficacy of phage-mediated killing of vancomycin-resistant *Enterococcus* (VRE) (123). Further, the immune response to infecting bacteria should also be considered, as that can affect both phage activity and clearance (124). Evaluating immunological responses to bacteriophages, such as cellular recruitment, cytokine responses, or antibody levels, is important in developing immunological criteria before patient enrollment in phage therapy.

## Discussion

Although filamentous bacteriophages cannot directly infect human cells, they have been shown to impact human health through their ability to alter several aspects of bacterial physiology, such as increased colonization (31) and biofilm formation (32), facilitation of horizontal gene transfer (52) and nutrient acquisition (33), and modulating host immune responses (35). However, our understanding of their biological significance as a vital component of our virome remains limited and is largely derived from studies involving a small number of filamentous phages associated with pathogenic bacteria (3, 31, 35, 64, 84). Two major themes emerge from studies examining the immunogenicity of filamentous bacteriophages. One is the complex tripartite interactions involving the filamentous phage, its bacterial host, and the mammalian immune system. In addition to influencing bacterial physiology, filamentous phages can directly interact with mammalian epithelial and immune cells, inducing inflammatory and antiviral signaling pathways that shape host responses to colonizing bacteria and phages (122). These studies highlight the need to further study these complex interactions, which could have far-reaching implications for the microbiome and overall human health (**Figure 2**). The second major theme is that mammalian cells exhibit differential immune responses to filamentous bacteriophages. The mechanism(s) underlying these differences remain unclear, but it may be due to recognizing a specific epitope on the surface of the bacteriophage (35). Very few studies have examined the specific components of bacteriophages that elicit cellular responses (35, 105, 117, 125). This could lead to future research examining the immunogenicity of specific bacteriophage proteins, elucidating further signaling pathways, and thereby obtaining a holistic understanding of filamentous bacteriophage immunogenicity. In summary, filamentous bacteriophages are an exciting, burgeoning area of research in the realms of microbiology and immunology. Further investigations will yield additional insights into their immunogenicity and potential effects on human health and physiology.

**Figure 2:**
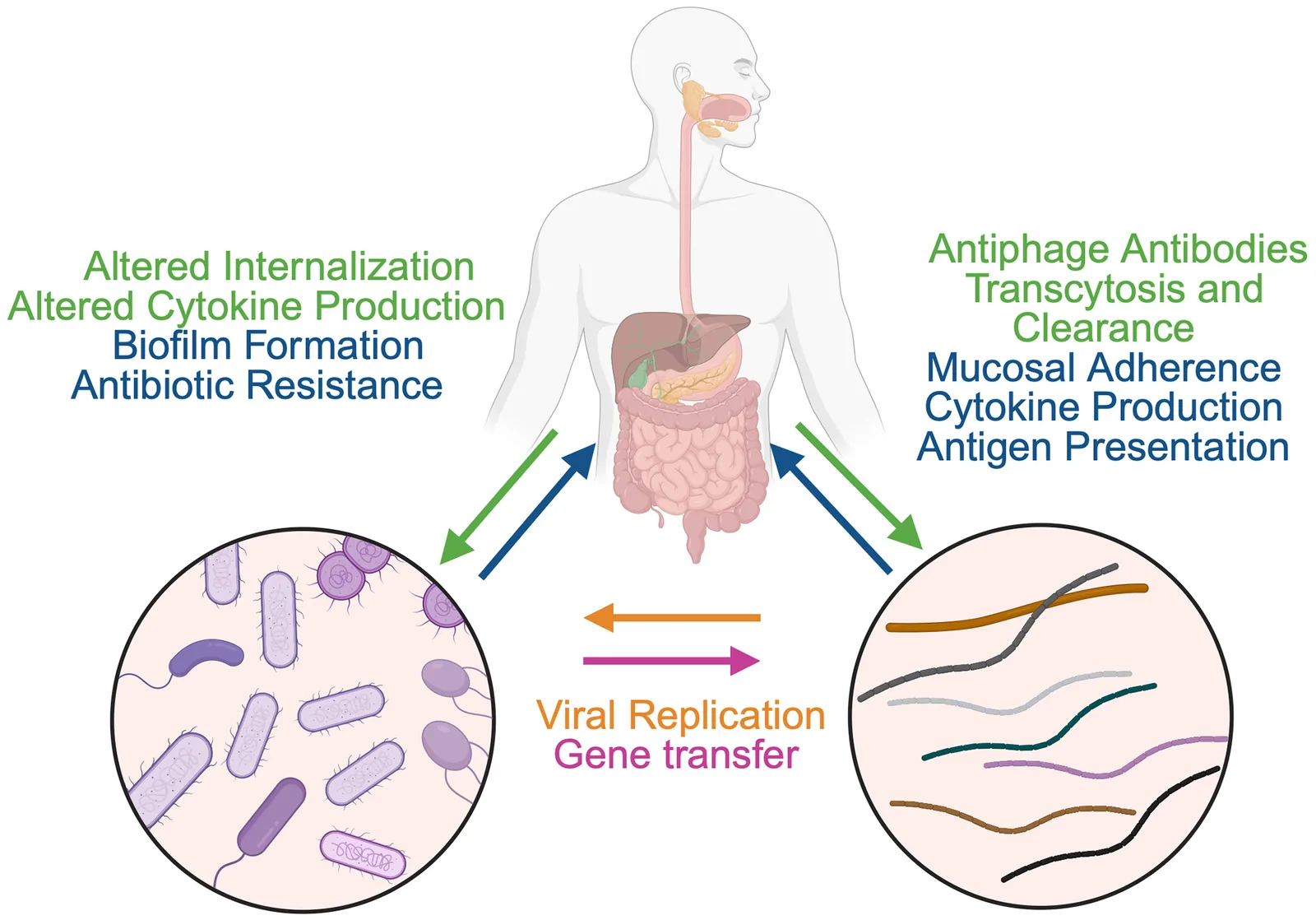
Tripartite interactions between filamentous bacteriophages, their host bacteria, and the human gut. The human microbiome is a complex consortium of bacteria, viruses, and fungi. Interactions between these individual components of the microbiome and human cells have been relatively well explored. However, the interplay between these organisms and how those dynamics impact human health is much less understood. This image highlights the interactions between filamentous bacteriophages and their host bacteria, and how these interactions, both directly and indirectly, shape intestinal immunity. The colors of the words and arrows match to indicate what each is contributing to the tripartite interaction. This image was adapted from Popescu et al. (126).
